# Evaluation of A Suicide Prevention Program in Switzerland: Protocol of A Cluster Non-Randomized Controlled Trial

**DOI:** 10.3390/ijerph16112049

**Published:** 2019-06-10

**Authors:** Stéphanie Baggio, Abbas Kanani, Neslie Nsingi, Marlène Sapin, Raphaël Thélin

**Affiliations:** 1 Division of Prison Health, Geneva University Hospitals & University of Geneva, Geneva 1205, Switzerland; stephanie.baggio@hcuge.ch; 2 Institute of Forensic Psychiatry, Department of Forensic Medicine, University of Bern, Bern 3012, Switzerland; 3 Association Stop Suicide, Geneva 1205, Switzerland; abbas@stopsuicide.ch (A.K.); neslie@stopsuicide.ch (N.N.); raphael@stopsuicide.ch (R.T.); 4 Swiss Center of Expertise in Social Sciences (FORS) & Swiss National Centre of Competence in Research “LIVES–overcoming vulnerability: life course perspectives”, University of Lausanne, Lausanne 1015, Switzerland; marlene.sapin@unil.ch

**Keywords:** evaluation, primary prevention, suicide

## Abstract

Suicide is a major public health concern, which disproportionally affects young people. Therefore, effective prevention strategies are needed, but there is a dearth of controlled trials on this topic. Our study will evaluate the effectiveness of a suicide prevention program in Switzerland, where data are scarce. It will test whether the prevention program (1) increases knowledge of suicide and awareness of suicidal risks, (2) provides resources to seek/offer help, (3) increases communication skills related to suicide, (4) increases coping skills, (5) is acceptable, and (6) reduces suicidal ideation and psychological distress. The project will be a single-center cluster non-randomized controlled trial designed to compare an intervention group benefitting from the suicide prevention program with a control group. The potential benefits include a better understanding and evaluation of suicide prevention programs, which may lead to improved primary and secondary prevention practices.

## 1. Introduction

Suicide is the second leading cause of death among young people aged 15 to 29 worldwide [1]. In this age group, the suicide rate is, per 100,000, 10.4 globally, 12.2 in Europe, and 8.5 in Switzerland, respectively. In Switzerland, suicide is the first cause of death among young people (28% of all deaths are attributable to suicide) [2]. Therefore, it constitutes a major public health issue and there is a crucial need for effective prevention strategies. Development of evidence-based suicide prevention programs should be a health policy priority. Reducing adolescent suicide is challenging because young people with suicide ideation rarely seek help [3]. Besides, few programs are evidence-based, so empirical studies with a sound methodology are needed to provide further indication of the effectiveness of the existing preventive programs [4,5]. The paucity of controlled trials designed to evaluate preventive intervention programs has recently been pointed out [6,7,8].

Despite the lack of reliable empirical studies evaluating them, several suicide prevention strategies have been developed at both societal and individual levels. They aim to reduce access to lethal means of suicide, to modify how media report suicide, to reduce psychiatric risk factors (e.g., promoting mental health and targeting at-risk groups) and to promote psychosocial interventions (e.g., reducing discrimination and isolation, promoting social support) [9]. One efficient prevention program is universal school-based interventions, such as the Youth Aware of Mental Health Program [10]. This kind of program usually uses interactive workshops including lectures about mental health and suicide, role-play sessions, and paper supports (e.g., booklets). Such programs are designed to raise awareness of suicide and risk factors to dispel myths about suicide, promote help-seeking behaviors, and develop adolescent coping skills. They are more effective than other school-based interventions to reduce suicide attempts and severe suicidal ideation [11]. Most published studies relied on school-based programs because they constitute the most effective way to reach young people. However, these interventions miss young people who are no longer enrolled in school, which is likely for those who are transitioning to adulthood. Therefore, none of the previous studies evaluated the full spectrum of possible interventions to prevent suicide among young people [7].

Prevention programs should be culturally sound. Indeed, attitudes toward suicide can vary widely between countries and regions and the way in which each community considers and experiences suicide should be taken into account. To our knowledge, no evaluation of suicide programs in school and community settings have been published in Switzerland. Even if there have been cantonal suicide prevention programs, the first Swiss national program on suicide prevention was created in 2016 [12]. In the French-speaking part of Switzerland, *Stop Suicide* delivers workshops for suicide prevention. The main aim of the workshops is to raise awareness of suicide and to provide resources to seek and offer help. More specifically, the covered topics include general information on suicidal behavior, ways to identify the risk factors and warning signals of suicide, myths and facts about suicide, how to ask questions, how to seek help, and how to provide support. The workshop has a duration of 90 min. The program includes one lecture, case examples with a group discussion, creation of a poster based on the group discussion, and an illustrated book. The facilitators include a staff member of *Stop Suicide*, who is trained to deliver it and to detect/manage suicidal risk, and a psychologist trained for suicide prevention and evaluation of suicidal risk, available during and after the workshop. It targets young people (males and females) aged between 14 and 29 in several different settings (e.g., schools, vocational schools, universities, and other higher education institutions, and settings for unemployed and disabled youths). *Stop Suicide* conducts an average of ten workshops per months (~1800 young people per year).

Therefore, this study will provide new insights into the effectiveness of this suicide prevention program in Switzerland, promoted by the association Stop Suicide (https://stopsuicide.ch). The project will test the following hypotheses:

**Hypothesis 1** **(H1).** The program increases knowledge of suicide and awareness of suicidal risks, including risk and protective factors.

**Hypothesis 2** **(H2).** The program provides resources to seek/offer help (e.g., whom young people can contact to seek help).

**Hypothesis 3** **(H3).** The program increases communication skills related to suicide (e.g., willingness and confidence to talk about suicide).

**Hypothesis 4** **(H4).** The program increases coping skills and young people are more able to manage stress related to adverse life events.

**Hypothesis 5** **(H5).** The program is acceptable (i.e., enjoyable and worthwhile for participants).

**Hypothesis 6** **(H6).** The program reduces suicide-related behaviors (suicidal ideation) and psychological distress.

## 2. Materials and Methods

### 2.1. Design

This study is a single-center cluster non-randomized controlled trial designed to compare an intervention group benefiting from the suicide prevention program with a control group. The study includes follow-ups at one and three months after the baseline assessment.

### 2.2. Participants

The study will take place in different settings in the French-speaking part of Switzerland. Workshops will take place in schools (classes) and community settings (associations, vocational schools, and homes/residential centers). We will recruit clusters from these settings. All young people are eligible to participate in the study with the following exclusion criteria: (1) Being under 14 years of age and (2) not being able to communicate in French.

### 2.3. Procedures

We will propose the study to all schools and community settings in which *Stop Suicide* delivers suicide prevention programs. We plan to rely on a random assignment to the intervention group or the control group. Participants from the control group will participate in the workshop at the end of the project. However, the final design cannot be considered as a pure randomized controlled trial since some schools or community settings may refuse to participate in the study before the workshop.

The first visit will start with the explanation of the study. Then, young people who consent to participate will complete the self-reported questionnaire (baseline assessment) (max. 20 min). 

For the intervention group, the suicide prevention program will be delivered right after to all young people, including those who did not consent to participate in the study. This one-and-a-half-hour universal program will be given in classes (or community settings) by the staff of *Stop Suicide*, who is trained to deliver it. For the control group, the first visit stops after the completion of the self-reported questionnaire.

The second visit will take place one month after the intervention (first follow-up). It will include the same questionnaires (excepted socio-demographic variables) as in the baseline assessment (max. 20 min).

The third visit will occur three months after the intervention (second follow-up). It will include the same questionnaires as in the first follow-up (max. 20 min). The control group will be delivered the workshop after the third visit.

Dissemination of information to different settings started in May 2019. Data collection will start in June 2019 and is planned to end in June 2020.

### 2.4. Measures

The whole French questionnaire is available in Supplementary Materials.

*Knowledge and awareness of suicide (H1)*. We will use seven items from the Literacy of Suicide Scale (LOSS) [13] to assess an increase in suicide knowledge and awareness of suicidal risks (five items of the original twelve-item scale are not discussed during the workshop). The items are assessed as true or false, leading to a total score of correct responses between 0 and 7 (Cronbach alpha = 0.77).

*Seek/offer help (H2)*. We will use the six items used in Bailey et al. [14] and a five-point scale to assess knowledge of what to do in case of suicidal risk and how to seek help. We will also use six self-developed items and a five-point scale to assess knowledge of where to seek help in the case of suicidal risk (e.g., “I know where to go for help if I have suicidal thoughts”, “I know a phone number to call for help”).

*Social network (H2)*. In addition, we will ask the participants to answer the two following questions:

1. With which classmates do you feel comfortable speaking about problems?
2. With which person outside the class do you feel comfortable speaking about problems?

For classmates, we will ask for first and last names in order to be able to create the complete class network. For persons outside the class, the participants will be invited to provide a first name and their relationship with this person (e.g., mother, father, sibling, friend, neighbors). We will be able to draw personal networks from this question and disentangle the family network from the peer network.

*Willingness and confidence to talk about suicide (H3)*. We will use the eight items used in Bailey et al. [14] on a five-point scale (Cronbach alphas: Willingness: 0.76, confidence: 0.60).

*Coping skills and management of adverse life events (H4)*. Two subscales from the COPE inventory [15] will be used: Seeking social support for instrumental reasons (Cronbach alpha = 0.75) and seeking social support for emotional reasons (Cronbach alpha = 0.85). Each subscale has four questions assessed on a four-point scale.

*Acceptability (H5)*. Acceptability will be assessed using six questions from Bailey et al. [14] (five-point scale).

*Suicidal ideation (H6)*. We will use two items related to the presence of suicidal ideation of the Columbia-Suicide Severity Rating Scale (C-SSRS, Cronbach alphas between 0.73 and 0.95), including a lifetime and past-month screen of suicidal thoughts (yes/no answers) [16].

*Psychological distress (H6)*. We will use the six items of the Kessler Psychological Distress Scale (K-6) [17,18] to assess psychological distress (five-point scale).

*Sociodemographic variables*. We will assess gender, age, type of school, and parental level of education.

### 2.5. Analytical Strategy

#### 2.5.1. Sample Size Calculation

With a confidence of 95%, a power of 80%, an expected difference between the two groups of 1, and a variance of 6, a sample of *n* = 190 (95 participants in each group) is needed to detect a statistically significant difference. We plan to include *n* = 300 participants (*n* = 150 in each group).

#### 2.5.2. Data Analysis

The project will use an intention-to-treat analysis. For all analyses, we will use three-level mixed-effect models, with measures nested in participants, who are themselves nested in classes/community settings. To provide a better overview of the benefits of the program, we will also use a per-protocol analysis. For all statistical analyses, we will use a two-sided α = 0.05. We will use a Bonferroni–Holm adjustment for multiple testing. When possible, the measures of effect size will be used to provide an estimation of the clinical significance of the findings. Statistical software will include Stata 15, SPSS 25, and R 3.4.3.

*Preliminary analyses:* We will examine the variables for distributional properties, correlations, and patterns of dropouts and missing data. We will compute descriptive statistics for each item and also for each scale. We will also check the reliability of the scales, especially for those that have not been described in previous studies. We will test differences between the intervention and the control group at baseline (socio-demographics and outcome variables) to confirm that the groups are comparable, especially if we cannot achieve a randomized allocation.

*H1. Increase in suicide knowledge and awareness of suicidal risks:* We will compare the mean scores of the literacy of suicide scale and the knowledge of what to do in case of suicidal thinking between the intervention and control groups.

*H2. Increase in skills related to how to seek/offer help:* We will compare the mean scores of help-seeking between the intervention and control groups.

*H2. Social networks:* Social networks of peers will be analyzed using exponential random graph models. Social networks of people outside the class are personal networks, rather than closed networks, so it will not be possible to use exponential random graph models. We will derive different variables (family support, friends’ support, other) to see whether there is an increase over time. We will explore different research questions: Is the density of social support related to suicidal thoughts and psychological distress? Is the diversity of social support related to a reduced level of suicidal thoughts and psychological distress? Does the program increase the density of social support?

*H3. Increase in skills related to the willingness and confidence to talk about suicide:* We will compare the mean scores of the willingness to talk about suicide and the confidence to talk about suicide between the intervention and control groups.

*H4. Increase in coping skills and management of adverse life events:* We will compare the mean scores of the coping subscales (and whole scale) between the intervention and control groups.

*H5. Program acceptability:* We will compute descriptive statistics of the six questions on program acceptability and of the mean score for the intervention group.

*H6. Reduction of suicidal ideation and psychological distress:* We will compare the mean scores of the suicidal ideation scale and the profile of mood states between the intervention and control groups.

In H1–H6 analyses, we will investigate the longitudinal associations (i.e., predicting the mean score at follow-up according to the group and the baseline level of the outcome variable), controlling for socio-demographic variables and other potential confounders assessed in the study. We will also consider using latent growth curve modeling.

Finally, we will compute the correlations between outcomes (knowledge, awareness, skills). Other analyses may involve subgroups for whom the intervention works better (e.g., according to socio-demographic variables, coping skills, psychological distress).

## 3. Expected Results and Conclusions

This project will provide valuable information about the efficacy of a suicide prevention program in Switzerland, where empirical evidence of the benefits of a suicide prevention program is missing. Potential benefits include a better understanding and evaluation of suicide prevention programs, which may lead to improvements in prevention practices, for both primary and secondary prevention. In addition, the study will provide evidence of the efficacy of such programs in community settings, in which data are scarce. It may also help to identify at-risk persons and groups.

Our findings will be published in leading peer-reviewed journals related to public health, adolescents’/young adults’ health, and mental health. We also intend to disseminate our results in different school/community settings. Data will be available upon request to the first author to investigate secondary research questions.

## Supplementary Materials

The following are available online at https://www.mdpi.com/1660-4601/16/11/2049/s1.
