# Supplementary material for: Evaluation of A Suicide Prevention Program in Switzerland: Protocol of A Cluster Non-Randomized Controlled Trial
Source: Int J Environ Res Public Health. 2019 Jun 10;16(11):2049. doi: 10.3390/ijerph16112049 (PMC6603986; doi:10.3390/ijerph16112049)
Supplement: Supplementary file 1 [file ijerph-16-02049-s001.pdf]

QUESTIONNAIRE

Merci d'avoir accepté de répondre à ce questionnaire.

Ce questionnaire porte sur le suicide et sur ton propre bien-être. Nous te demandons de cocher la case correspondant à ton choix pour chaque ligne. Merci de répondre à toutes les questions.

Identifiant : _ _ _ _ _ _ _ _ _ _ _ _
Date : _ _ / _ _ / _ _ _ _

Les questions suivantes portent sur ce que tu sais du suicide.

A. D'après toi, les énoncés suivants sont-ils vrais ou faux ?	Vrai	Faux
1. Les personnes qui ont des idées suicidaires ne devraient pas en parler à autrui.		
2. Consulter un psychiatre ou un psychologue peut aider à prévenir le suicide.		
3. Parler du suicide augmente toujours le risque suicidaire.		
4. Une personne suicidaire restera toujours suicidaire et aura des idées suicidaires.		
5. Très peu de personnes ont des idées suicidaires.		
6. Les personnes qui parlent de suicide se suicident rarement.		
7. Les personnes qui veulent tenter de se suicider peuvent rapidement changer d'avis.		

B. Es-tu d'accord avec les affirmations suivantes ?	Pas du tout d'accord	Pas d'accord	Ni d'accord, ni en désaccord	Plutôt d'accord	Tout à fait d'accord
1. J'ai les connaissances nécessaires pour reconnaître chez moi les signaux d'alerte du suicide.					
2. J'ai les connaissances nécessaires pour reconnaître les signaux d'alerte du suicide chez les autres.					
3. J'ai les connaissances nécessaires pour parler du suicide avec les autres.					
4. J'ai les connaissances nécessaires pour demander directement à quelqu'un s'il/elle pense au suicide.					

	Pas du tout d'accord	Pas d'accord	Ni d'accord, ni en désaccord	Plutôt d'accord	Tout à fait d'accord
5. J'ai les connaissances nécessaires pour chercher de l'aide en cas d'idées suicidaires.					
6. Je me sentirais prêt à parler de suicide avec les autres.					
7. Je me sentirais prêt à demander directement à quelqu'un s'il/elle pense au suicide.					
8. Je me sentirais prêt à chercher de l'aide en cas d'idées suicidaires.					
9. Je me sentirais confiant à l'idée de reconnaître chez moi les signaux d'alerte du suicide.					
10. Je me sentirais confiant à l'idée de reconnaître les signaux d'alerte du suicide chez les autres.					
11. Je me sentirais assez confiant à l'idée de parler du suicide avec les autres.					
12. Je me sentirais assez confiant à l'idée de demander directement à quelqu'un s'il/elle pense au suicide.					
13. Je suis assez confiant à l'idée de demander de l'aide en cas de pensées suicidaires.					
14. Je serais prêt à rechercher de l'aide pour un problème tel que des pensées suicidaires.					
15. Je sais où aller chercher de l'aide si j'ai des idées suicidaires.					
16. Je sais où aller chercher de l'aide si un(e) proche a des idées suicidaires.					
17. Je sais vers quel adulte chercher de l'aide à l'intérieur de mon établissement d'étude/travail.					
18. Je connais un numéro de téléphone à appeler pour demander de l'aide.					
19. Je sais quel professionnel peut m'aider ou aider un(e) proche.					
20. Je sais où trouver les numéros de téléphone et/ou les adresses des ressources d'aide.					

C. Avec quels camarades de ta classe te sens-tu à l'aise pour parler de tes problèmes ?
Tu peux nommer autant de camarades que tu veux, en indiquant leurs noms et prénoms.
(Les réponses que tu fais sont confidentielles et personne à part les personnes de Stop Suicide en charge de l'étude ne verront les noms et prénoms que tu indiques ici.)

D. Avec quels autres proches à l'extérieur de la classe te sens-tu à l'aise pour parler de tes problèmes ?
Indique le prénom de la personne et qui elle est pour toi (père, mère, frère, sœur, cousin(e), grand-mère, grand-père, ami/camarade en dehors de l'école, voisin, médecin, ligue d'aide, association, etc.)
(Les réponses que tu fais sont confidentielles et personne à part les personnes de Stop Suicide en charge de l'étude ne verront les personnes que tu indiques ici. Pour les lignes d'aide et associations, tu peux mentionner le ou les prénoms des personnes avec qui tu es à l'aise pour parler de tes problèmes).
Exemples :
Sarah, meilleure amie
Daniel, grand frère

Les questions suivantes portent sur toi, ce que tu ressens et ta façon de gérer les problèmes.

E. Comment te sens-tu et que fais-tu en cas de situation stressante ?	Jamais	Quelques fois	La plupart du temps	Toujours
1. J'essaie de trouver une stratégie afin de savoir quoi faire.				
2. Je fais un plan d'action.				
3. Je réfléchis sérieusement aux mesures à prendre.				
4. Je réfléchis à la meilleure façon de régler le problème.				
5. Je demande aux gens qui ont eu des expériences similaires, ce qu'ils ont fait.				
6. J'essaie d'obtenir des conseils de quelqu'un sur ce qu'il faut faire.				
7. Je parle à quelqu'un afin d'en savoir plus sur la situation.				
8. Je parle à quelqu'un qui pourrait faire quelque chose de concret pour résoudre mon problème.				
9. Je parle de ce que je ressens à quelqu'un.				
10. J'essaie d'obtenir du soutien émotionnel de mes ami-e-s ou de mes proches.				
11. Je parle de mes sentiments avec quelqu'un.				
12. Je reçois de la sympathie et de la compréhension de quelqu'un.				

F. Au cours des quatre dernières semaines, à quelle fréquence t'es-tu senti(e)…	Jamais	Rarement	De temps en temps	Souvent	Tout le temps
1. Nerveux/nerveuse ?					
2. Désespéré(e) ?					
3. Agité(e) ou ne tenant pas en place ?					
4. Si déprimé(e) que plus rien ne pouvait te faire sourire ?					
5. Comme si tout était un effort ?					
6. Bon(ne) à rien ?					

G. Au cours de ta vie…	Oui	Non
1. As-tu souhaité être mort ou souhaité t'endormir et ne pas te réveiller ?		
2. As-tu déjà pensé à te suicider ?		
Au cours des quatre dernières semaines…		
3. As-tu souhaité être mort ou souhaité t'endormir et ne pas te réveiller ?		
4. As-tu déjà pensé à te suicider ?		

H. Quelques informations sur toi...

1. Quel âge as-tu ? ________ ans

2. Es-tu :

☐ une fille ☐ un garçon ☐ neutre

3. Dans quel établissement es-tu ?

☐ Ecole obligatoire
☐ Ecole de commerce
☐ Ecole de culture générale
☐ SeMo
☐ Orif
☐ Foyer
☐ Ecole professionnelle
☐ Gymnase, collège ou lycée
☐ Autre

4. Quel est le niveau d'éducation le plus élevé de tes parents ?

☐ Ecole obligatoire
☐ Apprentissage
☐ Ecole de commerce / école de culture générale
☐ Haute école / Université

Pour finir, nous aimerions savoir ce que tu as pensé de l'atelier STOP SUICIDE.

I. Qu'as-tu pensé de l'atelier Stop Suicide ?	Pas du tout d'accord	Pas d'accord	Ni d'accord, ni en désaccord	D'accord	Tout à fait d'accord
1. As-tu apprécié l'atelier ?					
2. As-tu trouvé l'atelier perturbant ?					
3. Te sens-tu prêt(e) pour aider tes ami(e)s ou tes proches ?					
4. Penses-tu que tes ami(e)s se sentent prêt(e)s pour t'aider ?					
5. Est-ce que l'atelier en valait la peine pour les participant(e)s ?					
6. Recommanderais-tu l'atelier à un(e) ami(e) ?					

Merci beaucoup pour ta participation !
